# Supplementary material for: Slow-to-fast transition of giant creeping rockslides modulated by undrained loading in basal shear zones
Source: Nat Commun. 2020 Mar 12;11:1352. doi: 10.1038/s41467-020-15093-3 (PMC7067777; doi:10.1038/s41467-020-15093-3)
Supplement: Supplementary file 2 — Supplementary Information [file 41467_2020_15093_MOESM2_ESM.pdf]

Slow-to-fast transition of giant creeping rockslides modulated by undrained loading in basal shear zones

Agliardi et al.

<u>Supplementary Information</u>

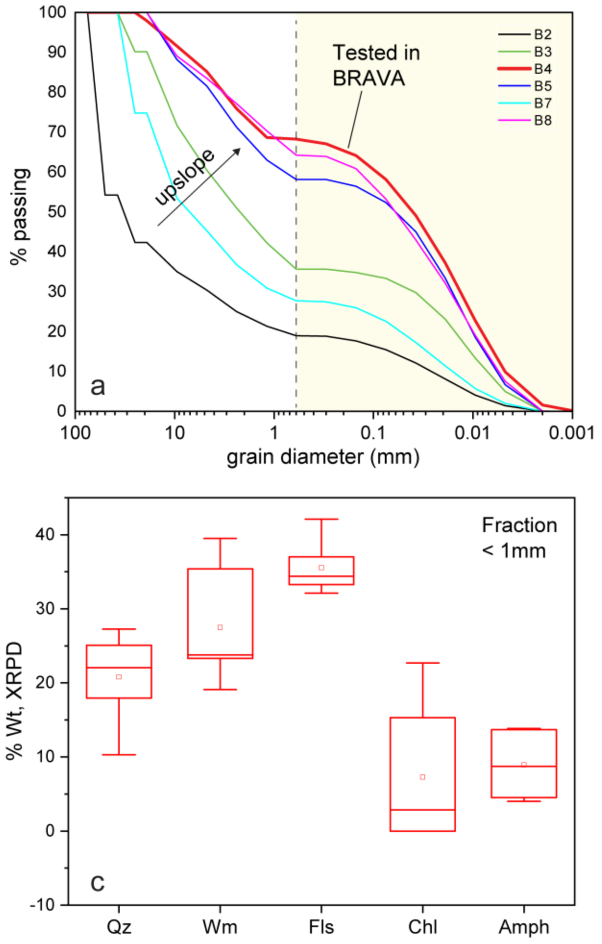

Supplementary Figure 1. Grain size and mineral compositions.

(a) Grain size distributions of cataclastic shear zone samples collected from the basal shear zone of the Spriana rockslide (locations in Fig. 1b). The yellow shade indicated the grain size fraction subjected to laboratory experiments; **(b,c,d)** mineral composition distributions (XRPD) of different gouge grain size fractions, showing the weak dependence of mineral composition on the choice of the grain size fraction subjected to laboratory experiments. Box plot elements: center line, median; square, mean; box limits, upper and lower quartiles; whiskers, 1.5x interquartile range; points, outliers.

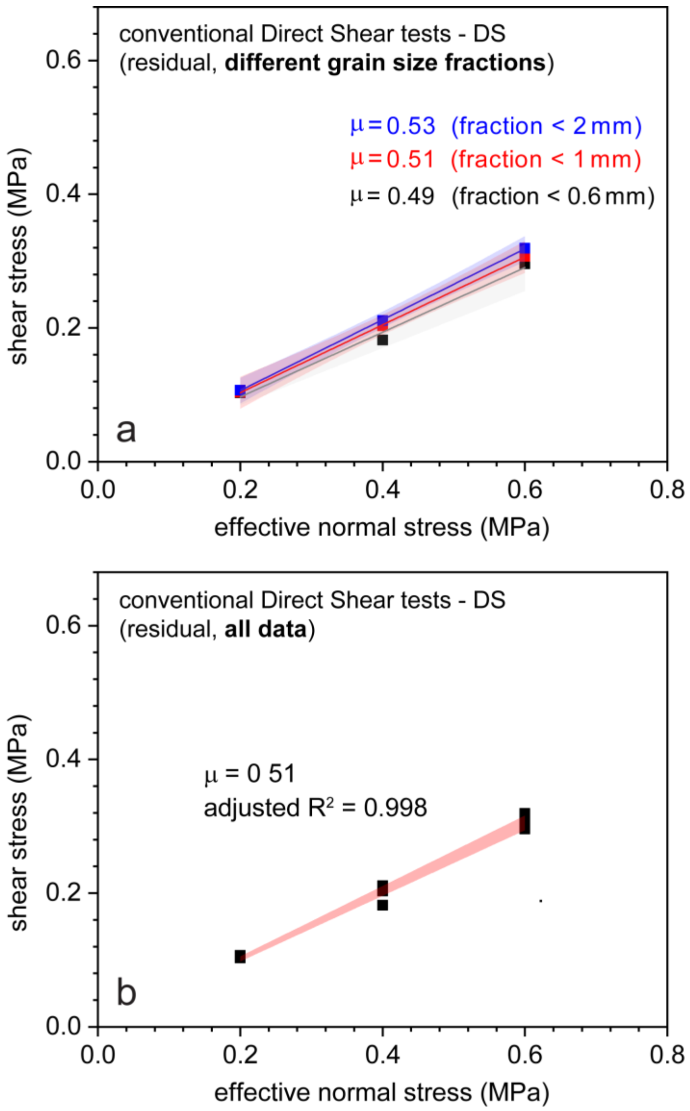

Supplementary Figure 2. Mohr-Coulomb envelopes.

Mohr Coulomb failure envelopes obtained on different gouge grain size fractions by conventional geotechnical direct shear tests, showing the weak dependence of frictional strength on the choice of the grain size fraction subjected to lab experiments: **(a)** envelopes obtained for individual grain size fractions; **(b)** envelope fit for the entire dataset. Shaded areas are 95% confidence bands of linear fit.

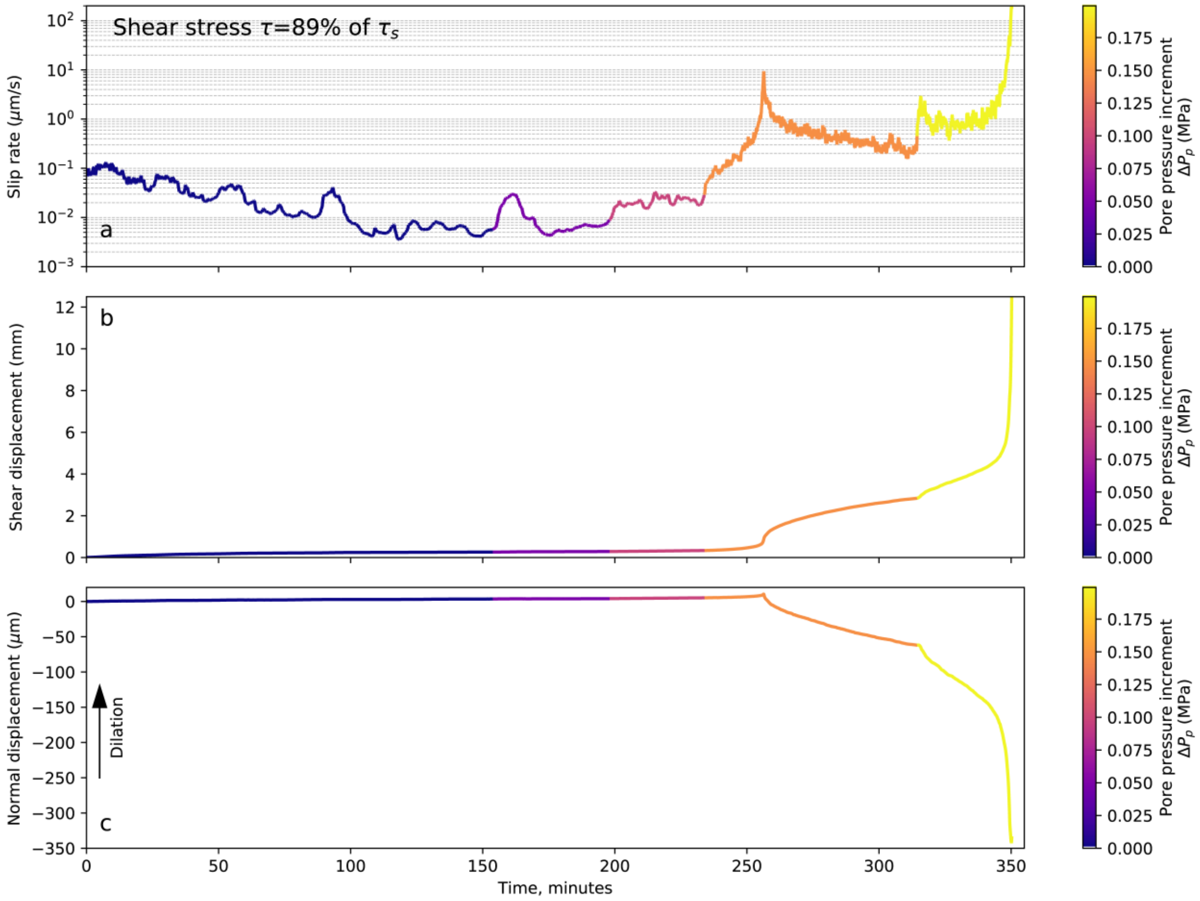

Supplementary Figure 3. Shear zone evolution during laboratory pore pressure-step creep experiment (τ=0.89 τ_s).

Experimental time series of **(a)** slip (shear displacement) rate; **(b)** cumulative shear displacement; **(c)** normal displacement (positive value: dilation).

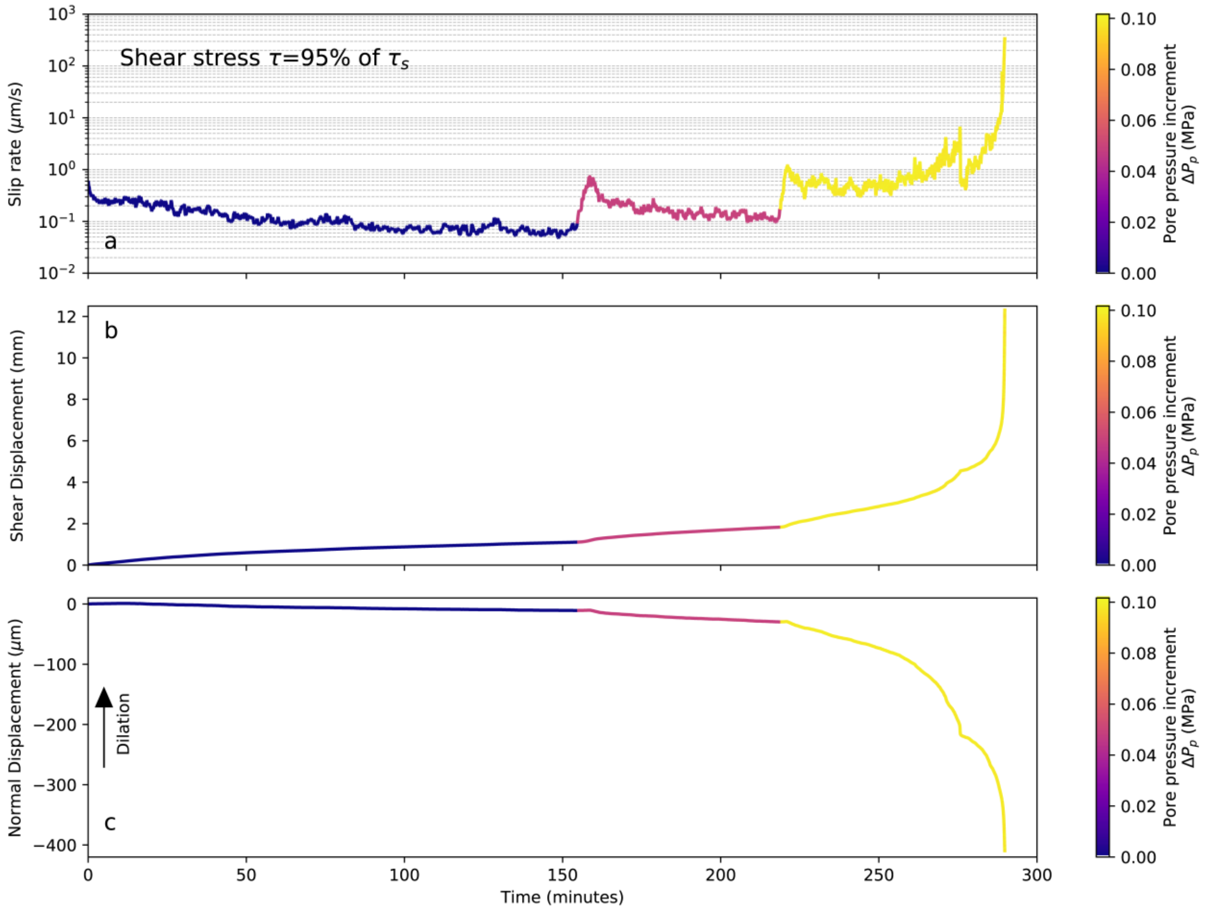

Supplementary Figure 4. **Shear zone evolution during laboratory pore pressure-step creep experiment (**τ**=0.95** τ_s**)**.

Experimental time series of **(a)** slip (shear displacement) rate; **(b)** cumulative shear displacement; **(c)** normal displacement (positive value: dilation).

Acceleration period	Total Duration (days)	Time to peak velocity (TV_P, days)	Peak velocity (V_P, μm/s)	Long-term velocity (V_{CII}, μm/s)
**1**	32	3	0.067	0.003
**2**	35	3	0.0185	0.002
**3**	60	2	0.022	0.0017
**4**	47	5	0.031	0.0015
**5**	47	6	0.012	0.0025
**6**	18	5	0.012	0.0023
**7**	150	15	0.013	0.004
**8**	79	11	0.023	0.004

Supplementary Table 1. Characteristics of selected rockslide acceleration periods (Fig. 1).

Variables	Dataset	# data	Spearman ρ	p-value	Perason r	p-value
**$T_{VP} – \Delta P_f$** (Fig. 5b)	Lab: τ_{mob} = 0.86	3	-0.5	0.67	-	-
	Lab: τ_{mob} = 0.89	4	-1	-	-0.93	0.07
	Lab: τ_{mob} = 0.95	**2**	-	-	-	-
	Rockslide	8	-0.53	0.17	-	-
**$V_P – \Delta P_f$** (Fig. 5c)	Lab: τ_{mob} = 0.86	3	1	-	0.997	0.046
	Lab: τ_{mob} = 0.89	4	0.8	0.2	-	-
	Lab: τ_{mob} = 0.95	**2**	-	-	-	-
	Rockslide	8	0.13	0.75	-	-
**$V_{CII} – \Delta P_f$** (Fig. 5d)	Lab: τ_{mob} = 0.86	3	1	-	0.928	0.24
	Lab: τ_{mob} = 0.89	4	1	-	0.912	0.08
	Lab: τ_{mob} = 0.95	2	-	-	-	-
	Rockslide	8	0.92	0.001	0.9	0.002

Supplementary Table 2. Correlation statistics for laboratory and rockslide data in Fig. 5.
