# Supplementary material for: Slow-to-fast transition of giant creeping rockslides modulated by undrained loading in basal shear zones
Source: Nat Commun. 2020 Mar 12;11:1352. doi: 10.1038/s41467-020-15093-3 (PMC7067777; doi:10.1038/s41467-020-15093-3)
Supplement: Supplementary file 3 — Source Data [file 41467_2020_15093_MOESM3_ESM.zip › NCOMMS-19-25861A_Supplementary-Data_table.docx]

| **Experiment** | **Effective stress (MPa)** | **Experimental procedure** | **Mobilized *τ***  **(% of *τ_s_*)** | **Fluid conditions** |
| --- | --- | --- | --- | --- |
| i455 | 2 | Velocity steps  1-300 |  | Room humidity |
| i456 | 2 | Velocity steps  1-300 |  | Water saturated - Pf=0 |
| b504 | *σ'_n_* = 2 -3- 4 *P_c_* = 2 *σ_n_* = 1-2-3 *P_f_* = 1 | Stable-sliding shear experiment |  | Pressurized water |
| b503 | *σ'_n_* = 2  *P_c_* = 2 *σ_n_* = 1 *P_f_* = 1 | Pore pressure-step creep experiment | *τ* = 95% | Pressurized water |
| b505 | *σ'_n_* = 2  *P_c_* = 2 *σ_n_* = 1 *P_f_* = 1 | Pore pressure-step creep experiment | *τ* =90% | Pressurized water |
| b502 | *σ'_n_* = 2  *P_c_* = 2 *σ_n_* = 1 *P_f_* = 1 | Pore pressure-step creep experiment | *τ* =86% | Pressurized water |
